# Expression of steroid and xenobiotic receptor in uterine carcinosarcoma, leiomyosarcoma and endometrial stromal sarcoma

**DOI:** 10.3892/ol.2012.1094

**Published:** 2012-12-28

**Authors:** XIAONI YUE, HIROKI UTSUNOMIYA, JUN-ICHI AKAHIRA, FUMIHIKO SUZUKI, KIYOSHI ITO, SATORU NAGASE, HIRONOBU SASANO, NOBUO YAEGASHI

**Affiliations:** 1 Departments of Obstetrics and Gynecology, Graduate School of Medicine, Tohoku University, Sendai 980-8574, Japan;; 2 Pathology, Graduate School of Medicine, Tohoku University, Sendai 980-8574, Japan;; 3 Department of Obstetrics and Gynecology, Fudan University, Shanghai 042465, P.R. China

**Keywords:** steroid and xenobiotic receptor, uterine sarcoma, immunohistochemistry, histological scoring

## Abstract

We analyzed the expression of the steroid and xenobiotic receptor (SXR) in human uterine sarcomas and evaluated its clinical significance. Forty-seven cases with archival specimens were examined for SXR expression using immunohistochemistry. All cases were scored using a semi-quantitative histological scoring (HSCORE) method. Specimens with a HSCORE >40 were regarded as SXR-positive. Various clinicopathological variables, including the expression status of estrogen receptor (ER)-α, progesterone receptor (PR) and Ki67 (MIB-1) were examined. The mean SXR HSCOREs of carcinosarcoma (CS) and leiomyosarcoma (LMS) were 9.13 and 23.6, respectively, and SXR-positive rates were 3 out of 24 (12.5%) and 4 out of 17 (23.5%), respectively. SXR was not detected in endometrial stromal sarcoma (ESS). In CS cases, significant differences were detected between the expression of SXR and age and disease stages. There was no significant correlation between SXR-positive status and either disease-free survival or overall survival. Our results support an association between SXR and malignant behavior. Our results show that overexpression of SXR may represent a useful marker to identify patients with advanced-stage CS. In addition, our results showed that SXR may aid in the diagnosis of uterine sarcomas.

## Introduction

Uterine sarcomas are rare, accounting for 3–7% of malignant diseases in the uterine corpus. They have been classified into three main histologic subgroups: carcinosarcoma (CS), leiomyosarcoma (LMS) and endometrial stromal sarcoma (ESS). Our previous study showed that five-year survival rates among 121 patients were 52.1, 61.3 and 68.4% for CS, LMS and ESS, respectively (1).

The standard therapy for uterine sarcoma is surgery; however, adjuvant chemotherapy is generally administered in the advanced stages of the disease. Previous studies have reported that the response rate for paclitaxel was 18% (2) and 19% for cisplatin (3). The poor response to chemotherapy reflects the drug resistance of uterine sarcoma.

SXR is expressed in the colon, intestine, lungs and kidneys, where it plays a vital role in the metabolism of endogenous substances, such as bile acids, hormones and vitamins (4,5). While paclitaxel is a key anticancer drug for CS, as well as for epithelial ovarian cancer, several studies have demonstrated that SXR agonists such as rifampicin depress the activity of paclitaxel (6,7) and induce cellular proliferation in cancers such as ovarian (6), endometrial (8) and breast cancer (9). Further research has confirmed that downregulation of SXR inhibits endometrial cancer cell growth and induces apoptosis (10). Cytochrome P450 3A4 (CYP3A4) has been shown to enhance SXR activity in an SXR knockout animal model (11,12). Although multiple drug resistance 1 (MDR 1) has not been found to induce the activation of SXR in ovarian cancer (6), this has been reported in breast (9) and endometrial cancer (13). We previously reported that SXR overexpression is a prognostic factor in epithelial ovarian cancer and represents a useful marker for identifying patients at high risk of recurrence or mortality (14). However, the status of SXR has not yet been investigated in uterine sarcomas. In this study we analyzed the status of SXR expression in CS and correlated the findings with various clinicopathological characteristics. In addition, we also examined ESS, LMS, benign leiomyoma and normal endometria for SXR expression and compared the findings with those in CS. Finally, we evaluated the correlation between SXR expression and the clinicopathological features of uterine sarcomas.

Generally, it is difficult to histologically differentiate between LMS and ESS. The expression of CD10 and α-smooth muscle actin (αSMA) is measured to aid in the diagnosis of ESS and LMS. Therefore, we investigated the expression of CD10 and αSMA as well as SXR and evaluated whether SXR expression has the capacity to be a diagnostic marker for uterine sarcomas.

## Materials and methods

### Patients and tissue specimens

Forty-seven patients with uterine sarcomas (6 ESS, 17 LMS and 24 CS), 5 patients with uterine leiomyoma and 5 patients with normal myometrium who underwent surgical treatment between 1993 and 2008 at Tohoku University Hospital (Sendai, Japan), were included in this study. Data including age, histological subtype, stage, residual tumor, metastasis, chemotherapy (TJ, pacilitaxel+carboplatin; IAP, fosfamide+doxorubicin+cisplatin), recurrence and clinical outcome were collected. Histologic subtypes were determined according to WHO criteria. This study was approved by the Ethical Committee of Tohoku University School of Medicine and informed consent was obtained from the patients.

Disease-free survival and overall survival were measured from the date of initial surgery to the date of recurrence and/or mortality, or the date of the last visit. Patients with recurrence were treated with surgical resection or platinum-based chemotherapy. For survival estimates, patients who were alive or lost to follow-up were censored in 2008. The median follow-up period was 14.3 months (range 1–61 months). All specimens were fixed in 10% formalin for 24 to 48 h, embedded in paraffin and cut into 3 *μ*m sections.

### Immunohistochemistry

Tissue sections were immunostained by the streptavidin-biotin method using a Histofine kit (Nichirei-Biosciences, Tokyo, Japan). The antibodies used in this study are listed in Table I. The immunohistochemistry (IHC) method used has been previously described (9). Briefly, after the sections were dewaxed and rehydrated, the sections were placed in target retrieval solution or citric acid buffer (2 *μ*M citric acid and 9 mM trisodium citrate dehydrate, pH 6.0) and autoclaved at 120°C for 5 min for antigen retrieval. For αSMA, the slides were digested with trypsin at 37°C for 30 min. The antigen-antibody complex was then visualized with 3,3’-diaminobenzidine (1 mM 3,3’-diaminobenzidine, 50 mmol/l Tris.HCL, pH 7.6 and 0.006% H_2_O_2_) and counter-stained with hematoxylin. Normal small intestine was used as a positive control for SXR. In order to distinguish between ESS and LMS, IHC for CD10 and αSMA was also performed.

### Immunohistochemical scoring system

All cases were scored by a semi-quantitative histological scoring (HSCORE) method. Immunostaining intensity for each specimen was classified as: 1 (none or weak staining), 2 (moderate staining) and 3 (strong staining). The HSCORE of each case was obtained by multiplying each intensity level with the corresponding percentage of positive cells using the following formula: HSCORE = Σ(I*LI) where I and LI represent the intensity and labeling index, respectively (9,10). The final scores ranged from 0 to 300. Specimens with a HSCORE >40 were regarded as SXR-positive, while a HSCORE <40 was regarded as SXR-negative. The LI was obtained for carcinoma cells as described by Sasano *et al*(15). Briefly, two of the authors (X.Y. and J.A.) independently evaluated at least 500 carcinoma cells microscopically. Immunostained slides were evaluated using a double-headed light microscope. Inter-observer differences were <5%.

### Statistical analysis

Student’s t-test was used to analyze the association of SXR HSCORE with nuclear receptor status and patient characteristics. Survival was analyzed using the Kaplan-Meier method. Spearman’s Rho was used for analysis of IHC results with regard to SXR expression and nuclear receptors or Ki67 antigen expression. Comparison of positive rates was undertaken using the χ^2^ test. Statview 5.0 software (SAS Institute Inc., Cary, NC, USA) was used for all statistical analyses. P<0.05 was considered to indicate a statistically significant difference.

## Results

**Figure 1:**
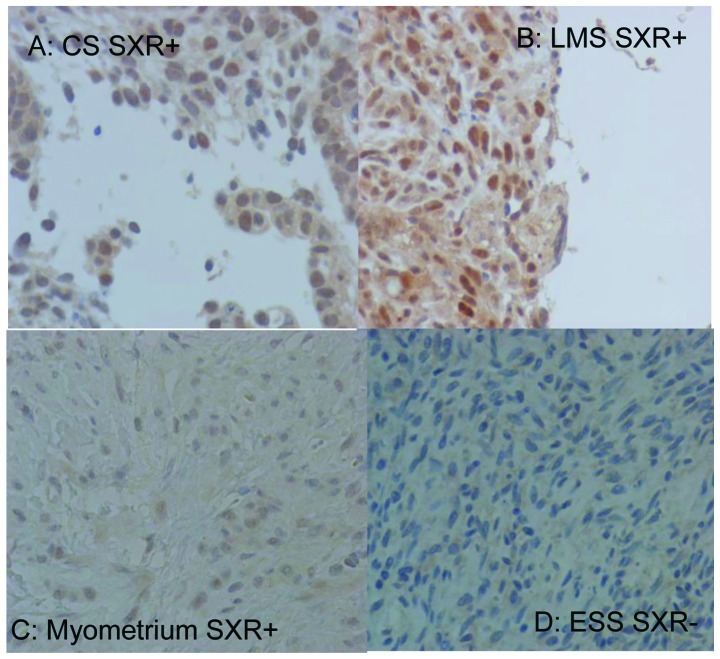
Immunohistochemistry for steroid and xenobiotic receptor (SXR) expression. Tissue sections of uterine sarcomas were immunostained by the streptavidin-biotin method using a Histofine kit. Original magnification, ×400. (A) SXR positive in carcinosarcoma (CS). (B) SXR positive in leiomyosarcoma (LMS). (C) SXR positive in normal myometrium. (D) SXR negative in endometrial stromal sarcoma (ESS).

SXR expression was detected in the nuclei of uterine sarcoma cells by IHC (Fig. 1A and B). An SXR HSCORE >40 was observed in 4 of 17 (23.5%) LMS cases and 3 of 24 (12.5%) CS cases. In normal myometrium, positive expression of SXR was observed in 1 of 5 specimens (Fig. 1C). No significant difference in SXR expression was observed between uterine sarcoma and normal myometrium (P=0.764), while no SXR expression was observed in ESS cases (Fig. 1D) and benign leiomyomas.

**Figure 2:**
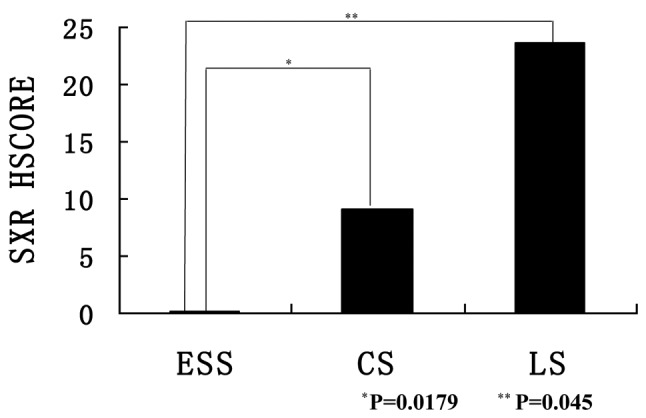
Comparison of steroid and xenobiotic receptor (SXR) HSCORE among uterine sarcomas. All cases were scored by a semi-quantitative histological scoring (HSCORE) method. Student’s t-test was used to analyze the association of SXR HSCORE. Results are expressed as the mean ± standard error (SE). ^*^P=0.0179 vs. carcinosarcoma (CS); ^**^P=0.045 vs. leiomyosarcoma (LS). ESS, endometrial stromal sarcoma.

The mean SXR HSCOREs for CS and LMS were 9.13 (range 0–57.2) and 23.6 (range 0–135.6), respectively. The degree of expression was higher in LMS than in CS. Significant differences in median SXR HSCOREs were observed between ESS and CS and ESS and LMS (Fig. 2; P=0.0179 and 0.045, respectively). The correlations between SXR expression and clinicopathological features were analyzed (Table II). There were significant differences between SXR expression and stage, age and Ki67 expression in CS (P<0.05), while no significant differences were identified in LMS.

We analyzed the association between SXR expression and survival rate or clinical stage in CS. SXR-positive cases were detected in 3 of 9 (33.3%) advanced-stage patients with CS, whereas there were only 3 of 17 (17.6%) patients with CS who were disease-free during the follow-up period. In CS patients who were SXR-positive, there was no significant correlation with regard to survival (data not shown). Expression of ERα and PR was not significantly associated with disease-free survival or overall survival. Spearman’s Rho analysis showed that there was a statistically significant correlation between the HSCORE and Ki67 expression levels in CS (r=0.474, P=0.0230). The positive rates for CD10 were 23.5 and 100% in LMS and ESS, respectively (P=0.0011). The positive rates for αSMA were 58.8 and 66.7% in LMS and ESS, respectively, which were not significantly different.

## Discussion

Gupta *et al* found that SXR activation induced cell proliferation and drug resistance in ovarian cancer cells (6). In addition, SXR expression was also detected in the normal endometrium, in the proliferative and secretory phases (6,16). Other studies have reported SXR expression in normal and cancer tissues from the liver, breast and uterus (6,17,18). In our present study, SXR expression was detected in LMS and CS, but not in ESS. The percentage of cases with advanced-stage CS with positive SXR was higher than the percentage observed in the early stages, and a significant correlation between SXR expression and stage was found for CS (P<0.05).

It has been reported that TJ chemotherapy is effective for CS (19). However, we did not find an association between SXR expression and the efficacy of chemotherapy in uterine sarcomas. The poor response to chemotherapy reflects drug resistance in uterine sarcomas. In endometrial cancer, CYP3A4 and MDR1 activity were induced by the activation of SXR (8). We previously reported that SXR is a prognostic factor in epithelial ovarian cancer and may represent a useful marker for identifying patients at risk of recurrence or mortality (14). SXR is induced by paclitaxel, which is a major anti-cancer drug for CS as well as epithelial ovarian cancer. We investigated the correlation between SXR expression and CS; however, there was no significant correlation observed between SXR-positive status and both disease-free survival and overall survival. It has been reported in mice that SXR has two isoforms, SXR1 and SXR2 (20). These results suggest that the expression of SXR isoforms differs in different organs. The role of SXR isoforms in CS may be different from that in epithelial ovarian cancer. Further investigation is needed to clarify the status of SXR isoforms in human uterine sarcomas.

This study showed that there is a significant correlation between SXR-positive status and stage and metastasis in uterine sarcomas. In CS, SXR expression was also significantly related to stage and Ki67 expression. Our results support an association between SXR expression and malignant behavior. Overexpression of SXR may aid in identifying patients at an advanced stage of CS.

The assessment of SXR expression by the HSCORE method incorporates the intensity of staining and the LI (percentage of stained cells for each intensity level). Therefore, in comparison to a standard evaluation of immunohistochemical results by a 3-tier score (1), HSCORE provides a more accurate and homogeneous assessment of protein expression levels in individual cases. The expression levels using monoclonal nuclear antibodies for ERα, PR and Ki67 were 10–20% of the SXR LI in uterine sarcomas. The HSCORE range was 0–300 and the positive HSCORE range was inferred to be 30–60. Therefore, this suggests that a HSCORE of 40 is the threshold for identification of uterine sarcomas.

It is often difficult for pathologists to distinguish LMS from ESS histologically. The determination of CD10 and αSMA expression is employed to aid in the diagnosis of ESS and LMS. In this study, the positive rates for CD10 were 23.5 and 100% in LMS and ESS, respectively (P=0.0011). In addition, the expression of SXR in LMS was significantly higher than that in ESS, in which it was completely absent. Therefore, our study suggests that SXR may be used as a diagnostic marker to identify LMS and ESS.

This is the first study to evaluate the correlation between SXR expression and clinical outcomes in uterine sarcomas. Overexpression of SXR may be employed to identify patients at advanced stages. Further studies are needed to clarify the role of SXR in the biology of human uterine sarcomas. Understanding the mechanisms of SXR may aid in the development of chemotherapeutic regimens specifically designed against SXR and its target genes.

## Figures and Tables

**Table I:** Summary of primary antibodies used in this study.

Antibody	Source	Optimal dilution	Antibody retrieval
SXR (monoclonal)	Perseus proteromics (Japan)	1:400	Autoclave^a^
ERα (monoclonal)	Invitrogen (UK)	1:1	Autoclave^a^
PR (monoclonal)	Chemicon (USA)	1:50	Autoclave^a^
Ki-67 (monoclonal)	DAKO (Denmark)	1:100	Autoclave^a^
CD10 (monoclonal)	Nichirei (Japan)	1:1	Autoclave^b^
αSMA (monoclonal)	DAKO (Denmark)	1:300	Trypsin^c^

a Heated in an autoclave at 120°C for 5 min in citric acid buffer (2 *μ*M citric acid and 9 mM trisodium citrate dehydrate, pH 6.0).

b Heated in an autoclave at 120°C for 5 min in target retrieval solution.

c Digested with trypsin at 37°C thermostat for 30 min. SXR, steroid and xenobiotic receptor; ERα, estrogen receptor-α; PR, progesterone receptor; αSMA, alpha-smooth muscle actin.

**Table II:** Assocation of SXR HSCORE and clinicopathological features in leiomysarcoma and carcinosarcoma.

Clinicopathological features	Carcinosarcoma			Leiomyosarcoma		
	
	n	SXR HSCORE (range)	P-value	n	SXR HSCORE (range)	P-value
Age (years)						
≤50	3	0 (0)	0.017^a^	4	33.95 (0–135.8)	0.3064
>50	21	10.44 (0–57.2)		13	20.42 (0–120.2)	
Stage						
I–II	8	0 (0)	0.0352^a^	5	8.04 (0–40.2)	0.3716
III–IV	16	13.7 (0–57.2)		12	30.08 (0–135.8)	
Residual tumor						
Optimal	17	10.26 (0–57.2)	0.22	12	22.12 (0–120.2)	0.4201
Suboptimal	7	6.4 (0–44.8)		5	27.16 (1–135.8)	
Chemotherapy						
Yes	18	10.89 (0–57.2)	0.2034	5	29.08 (0–120.2)	0.7559
No	6	3.85 (0–23.1)		12	21.32 (0–135.8)	
Metastasis						
Yes	15	12.99 (0–57.2)	0.085	13	27.77 (0–135.8)	0.5062
No	9	2.7 (0–24.3)		4	10.05 (0–40.2)	
Ki67 (%)						
<15	3	0 (0)	0.017^a^	8	13.13 (0–79.8)	0.3799
≥15	21	10.44 (0–57.2)		9	32.91 (0–135.8)	
ERα						
Negative	19	8.53 (0–46.3)	0.3746	9	26.69 (0–120.2)	0.7734
Positive	5	11.44 (0–57.2)		8	20.13 (0–135.8)	
PR						
Negative	16	10.13 (0–46.2)	0.3523	8	20.13 (0–135.8)	0.7734
Positive	8	7.15 (0–57.2)		9	26.69 (0–120.2)	
Total	24	9.13 (0–57.2)		17	23.6 (0–135.8)	

a P<0.05, considered to indicate a statistically significant difference. SXR HSCORE, steroid and xenobiotic receptor histological scoring; ERα, estrogen receptor-α; PR, progesterone receptor.
